# Application of Aramid Nanofibers in Nanocomposites: A Brief Review

**DOI:** 10.3390/polym13183071

**Published:** 2021-09-11

**Authors:** Yangyang Fan, Zhihua Li, Junchao Wei

**Affiliations:** 1 School of Stomatology, Nanchang University, Nanchang 330006, China; Fanyy16655285545@163.com; 2 Jiangxi Province Key Laboratory of Oral Biomedicine, Nanchang 330006, China; 3 College of Chemistry, Nanchang University, Nanchang 330031, China

**Keywords:** aramid nanofiber, composite materials, blend, mechanical properties

## Abstract

The diameter of fibers is a critical factor in determining their final applications. When the diameter of aramid fibers changes from microns to nanoscale, its range of applications will be greatly extended. In this short review, the preparation of aramid nanofibers (ANFs) with diameters from ten nanometers to more than one hundred nanometers is introduced. Due to their excellent mechanical properties and their chemical and thermal stability, ANFs have been widely used as novel nanomaterials and composited with other materials, mainly for use in reinforced composites, energy storage, filtration and adsorption, biomedicine and electromagnetic fields. In this short review, the application of ANFs and their composites during the last 10 years is concisely summarized and a brief perspective on ANFs and their composites is also presented.

## 1. Introduction

Aramid fiber is a kind of aromatic polyamide fiber that possesses high temperature resistance, chemical corrosion resistance, fatigue resistance and super mechanical properties. For example, Kevlar fiber, which is made from para-aramid, has excellent mechanical properties, its tensile strength can reach about 3.6 GPa, and its modulus can reach about 120 GPa; thus, it has been widely used in the aerospace, military protection, the automotive industry and sporting goods fields [1,2,3]. Generally, the diameter of aramid fiber ranges from several to approximately ten microns [4]. The diameter of fibers may be a critical factor affecting its properties and final applications. Reducing the diameter of aramid fibers to a nanoscale may bring some excellent new properties, and the nanofibers may also work as fillers to blend with other materials and prepare functional composites. Great efforts have therefore been made to develop aramid nanofibers (ANFs).

Electrospinning is an efficient method to prepare various nanofibers and has been widely used in drug delivery and tissue engineering, amongst other applications [5]. However, a critical factor that can affect electrospinning is the solubility of electrospun materials. As for aramid fiber, due to the high crystallinity of aramid fibers and the strong hydrogen bonds between aramid molecules, it is much more difficult to prepare ANFs via the electrospinning method. Nevertheless, Yang’s group designed a top-down method with aramid fibers as the raw materials, dissolving them in dimethyl acetamide with LiCl to form an electrospinning solution, and obtained electrospun ANFs therefrom. However, in this experiment, a high temperature was required, and the diameters of fibers were about 69 to 180 nm [4]. Tuo’s group, by contrast, prepared ANFs via a bottom-up method, which used the polymerization of terephthaloyl chloride and p-phenylene diamine; meanwhile, methoxypolyethylene glycol (mPEG) was added during the polymerization process—an addition that can suppress the aggregation of liquid-crystal polymers in polyaramid chains. ANFs were then prepared by intensive shear and precipitating agents [6]. In 2011, Kotov used KOH and DMSO deprotonation methods to peel Kevlar fibers and obtained aramid nanofibers (ANFs) with a diameter of 5–30 nm and a length of 5–10 μm [7]. However, there were some drawbacks, such as low concentration, low efficiency and time consumption. Therefore, it is still a great challenge and of great importance to design new methods to obtain ANFs.

ANFs possess a high specific surface area and high aspect ratio and have shown excellent mechanical and stability properties. They have been widely used as novel nano-building blocks to prepare high strength films, aerogels and other functional materials [8,9,10]. In addition, ANFs can also be used as nano-fillers to blend with a variety of materials, such as polyvinyl alcohol (PVA) [11,12], polyurethane (PU) [13], polyacrylic acid (PAA) [14], polyethylene glycol (PEO) [15,16,17], carbon nanotubes (CNT) [18,19], graphene oxide (GO) [20,21,22,23], boron nitride nanosheets (BNNS) [24,25] and epoxy [26]. Previous work has demonstrated that ANFs and their nanocomposites have great potential applications in many fields [27,28,29], such as high-strength composites, energy storage, adsorption and filtration, biomedicine and electromagnetic fields.

In this short review, we briefly summarize the application of ANFs and their composites before discussing their existing problems and prospects. In drawing attention to the current state of research, we hope to generate interest and stimulate further work on ANFs so that they can be put to use in our daily life.

## 2. Application of Aramid Nanofibers

### 2.1. High-Strength Nanocomposites

**Figure 1:**
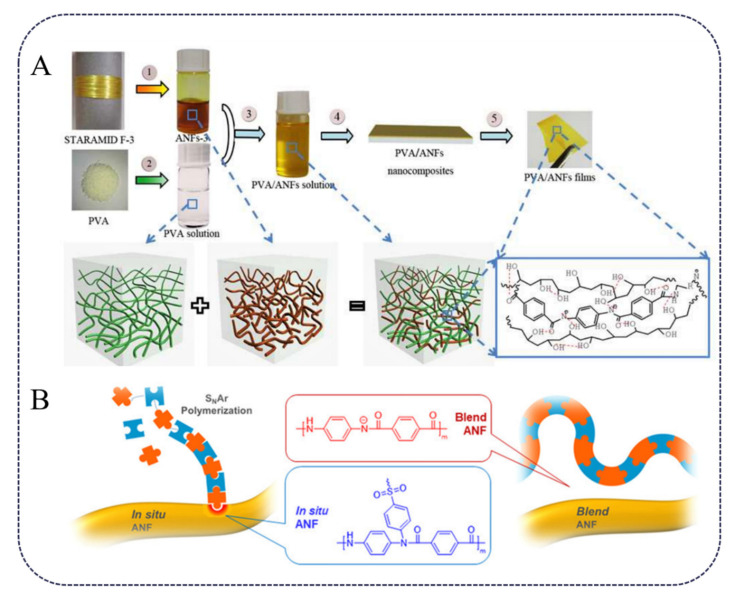
(**A**) Schematic illustration of interactions between PVA and ANFs via a simple solution casting method [11] (Copyright 2017 Elsevier). (**B**) Schematic illustration of the nanocomposite preparation by in situ and blending methods [30] (Copyright 2020, American Chemical Society).

Mechanical properties are the key factors that limit the application of composites. Improvements are restricted by nano-fillers and the interaction between nano-fillers and the polymer matrix. Hydrogen bonds and π–π interactions are easily formed between ANF molecular chains and other molecules, which can effectively enhance the interfacial adhesion between ANFs and matrix materials. Therefore, ANFs can be used as nano-filler to prepare nanocomposites with excellent mechanical strength and toughness. For example, PVA has been blended with ANFs and to effectively improve its mechanical properties. Guan [11] et al. prepared PVA/ANF composite membranes by a simple solution casting method (Figure 1A). The Young’s modulus of the film was 5.2 GPa, 18.2% higher than that of pure PVA film, and the tensile strength and toughness were, respectively, 79.2% and 148.8% higher than that of PVA. Kotov [12] et al. prepared a biomimetic PVA/ANF hydrogel with water content of 70–92%, and the tensile modulus, ultimate tensile strain, compressive strength and fracture toughness of the hydrogel reached 9.1 MPa, 325%, 26 MPa and 9200 J/m^2^, respectively. Yang [13] et al. used ANFs to reinforce polyurethane (PU), another kind of widely used polymer. Due to the multiple hydrogen bonding between ANFs and PU molecules, the mechanical properties of the PU/ANF composite were much better than those of PU, and its strength and Young’s modulus were 98.02 MPa and 5.275 ± 0.548 GPa, respectively. Kotov [14] et al. also prepared ANF/PAA (polyacrylic acid) composite films by a vacuum-assisted filtration method. When PAA content was 20%, the modulus of the film reached about 20 GPa.

**Figure 2:**
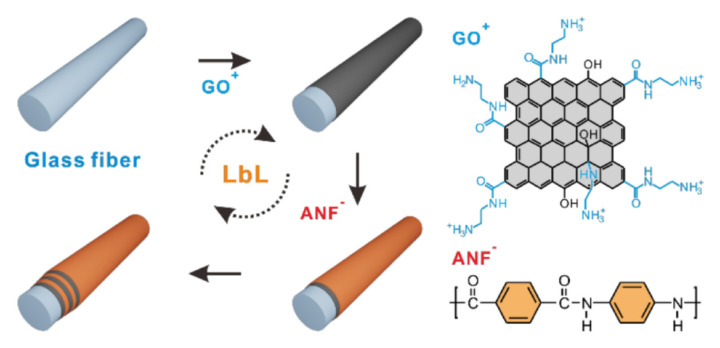
Schematic of the preparation of a positively charged graphene oxide (GO) and negatively charged aramid nanofiber (ANF) multilayer coating on a glass fiber via nanoscale blending layer-by-layer (LBL) assembly [32] (Copyright 2015, American Chemical Society).

The dispersion of nanofillers is one of the key factors influencing the properties of the composite material. The surface treatment of ANFs helps to improve its dispersion state and thus may improve its reinforcement effect. For example, polysulfone (PSU) was grafted onto an ANF surface via in situ S_N_Ar polymerization (Figure 1B), which maximized the dispersion of ANFs in the PSU matrix. Furthermore, only 0.15 wt. % nanofiller can make the tensile strength and toughness of PSU composites 1.6 times and 3.4 times higher than those of neat PSU, respectively [30]. ANFs can also be used to modify other components. For example, carbon nanotubes can be coated with ANFs by anodic electrophoresis, which can improve the bonding strength between carbon nanotubes and the epoxy matrix [31]. Park [32] et al. assembled ANFs and graphene oxide layers on the glass fiber, which improved the surface free energy and interfacial shear strength compared with those of the bare glass fiber (Figure 2). The interfacial adhesion interactions are highly adjustable and can be altered by changing the composition and structure of the coating.

By combining ANFs with different materials, composites with both strong mechanical properties and different functions can be obtained. The high mechanical strength lays the foundation for the application of composite materials in a variety of different environments, which gives ANF composites a bright application prospect in energy storage, adsorption filtration and biomedical applications, amongst other things.

### 2.2. Energy Storage

Energy storage has been a hot topic all over the world, but the insufficient performance of energy storage materials has limited the development of energy storage equipment. Over recent years, ANFs have been combined with energy storage materials to obtain strong materials that can be used as battery separators, solid electrolytes and electrode materials.

**Figure 3:**
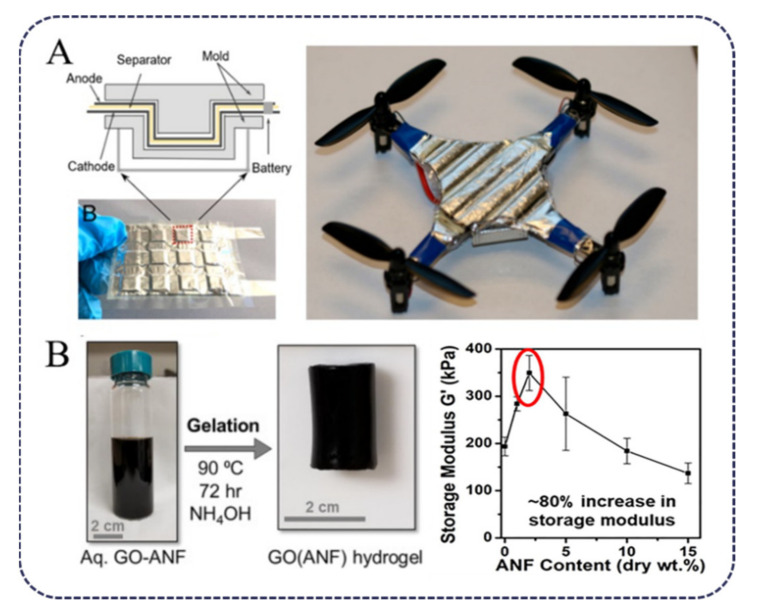
(**A**) Schematic of the mold used for plastic deformation studies and deformed shapes of Zn battery with solid-state biomimetic electrolyte PZB-931 for UAV [17] (Copyright 2019, American Chemical Society). (**B**) Sol–gel gelation results in formation of a GO(ANFx) hydrogel and comparison of storage modulus (G′) for all ANF loadings [23] (Copyright 2019, Elsevier).

Lithium-ion batteries are important energy storage devices, but there are still some safety issues, such as dendrites piercing the separator. Therefore, tuning the properties of the separator is an efficient method for improving the safety of lithium batteries. In 2015, Kotov [16] et al. prepared ANF/PEO (polyethylene oxide) ion-conducting membranes via the layer-by-layer (LbL) method. When (PEO/ANF)_200_ film (the number 200 represents the deposition cycles of LBL) as thin as 3 μm was used, the performance was comparable to that of commercial Celgard film with a thickness at least of 25 μm. Wang et al. [33] prepared an ANF/polyphenylene sulfide nonwoven composite separator, and ANFs regulated the porosity and enhanced electrolyte absorption of the film, resulting in better interfacial compatibility and superior ionic conductivity. ANFs can also be used in solid composite polymer electrolytes. ANF/PEO-LiTFSI (lithium bis(trifluoromethylsulfonyl)imide) composite polymer electrolytes (CPEs) can be obtained by solution casting method [34]. The hydrogen bond interaction between ANFs, PEO and LiTFSI can induce CPEs to form a 3D network, providing a place for the dissociation of LiTFSI and leading to the enhancement of electrochemical, mechanical and thermal stability. Zinc batteries have greater energy density and availability than lithium-ion batteries, but archetypal zinc batteries have the disadvantages of being inflexible, non-rechargeable and containing corrosive electrolytes. On the basis of research on lithium batteries, the development of solid electrolytes for Zn batteries seems to be an appropriate way to overcome the shortcomings. Kotov et al. [17] prepared a solid electrolyte of PEO/Zn(CF_3_SO_3_)_2_/BANFS (PZB) by adding Zn(CF_3_SO_3_)_2_ into the PEO/ANF network. The zinc battery made of PZB has a charging capacity and maintains coulomb efficiency of 96–100% after 50–100 charge–discharge cycles. The bionic solid electrolyte enables the battery to withstand elastic and even plastic deformation and makes the application of distributed capacitors possible (Figure 3A).

ANFs can be used to improve the mechanical and cycling properties of electrode materials. For example, (poly(3,4-ethylenedioxythiophene):poly(styrene sulfonate) (PEDOT:PSS) is a promising electrode materials; however, its low mechanical properties have limited its application. When ANFs were combined with PEDOT:PSS, a strong and flexible composite membrane ANF/PEDOT:PSS was prepared by vacuum filtration. Under bending and torsion conditions, the electrical conductivity of the composite film can still reach 534.2 S·cm^−1^ [35]. In addition, ANFs and polyaniline (PANI) were also used to fabricate flexible electrode materials. The energy storage and mechanical properties are achievable by adjusting the polymerization of PANI on an ANF substrate [36]. In 2017, a novel ANF/GO electrode was fabricated via the LBL method [22]. The chemically reduced electrodes exhibited capacitive charge storage with areal capacitances as high as 221 μF/cm^2^ (78 F/cm^3^) in the battery test. Therefore, both mechanical and electrochemical properties can be fully manipulated. The GO hydrogel electrode is a kind of graphene electrode material, which has a high specific surface area and high porosity, so it is widely used as a binder-free electrode for supercapacitors. However, its 3D architectures tend to have poor mechanical integrity. To solve this problem, in 2019, Micah J. Green’s group [23] fabricated an ANF/GO composite hydrogel electrode. Due to hydrogen bonds and π–π stack interactions between ANFs and GO, the shear modulus of the composite hydrogel with only 2% ANFs increased by 80% that of the GO hydrogel (Figure 3B).

The introduction of ANFs with their high strength, corrosion resistance and high temperature resistance helps to eliminate the performance defects in energy storage materials. By simply adjusting the composition of the composite, a balance of mechanical and electrical properties of materials can be obtained, which provides a new possibility for the further development and practical application of energy storage equipment.

### 2.3. Adsorption and Filtration

**Figure 4:**
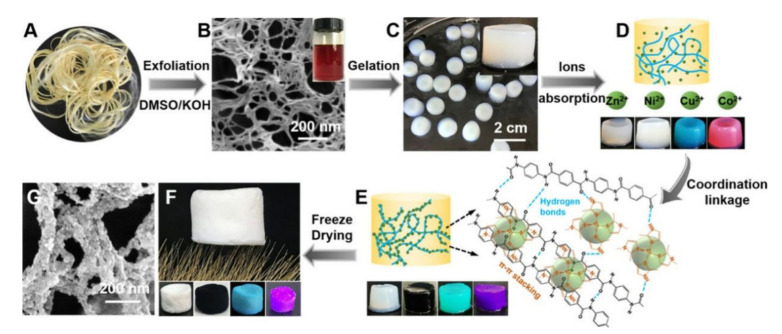
Pathway followed to fabricate MOF hybrid aerogels [40] (Copyright 2020, American Chemical Society).

With the rapid development of industry, water pollution has been a serious problem. Wastewater contains various harmful impurities, such as heavy metal ions and organic dyes, which cause serious harm to the biological and natural environment. Adsorption and filtration are commonly used methods for wastewater treatment, and, thus, various types of materials, such as fiber, membrane and aerogel, have been designed to treat polluted water [37,38,39]. ANFs, as an important polymer material, showed a high solvent tolerance and are an efficient candidate for water treatment. Wu et al. [40] prepared a metal–organic framework (MOF) incorporated with ANF aerogel and obtained a mixed aerogel (Figure 4), which exhibited superior adsorption performance for organic dyes from aqueous solution efficiently and continuously (e.g., 113.8 mg/g for methyl violet and 107 mg/g of rhodamine B, high flux (620 L/(h·m^2^·bar) at a thickness of ~0.87 mm). In addition, ANFs with special functional groups can also be used to detect metal ions in water via the special absorption ability of ANFs with respect to metal ions. For example, benzimidazole-containing ANF (B-ANF) can induce aggregation of many heavy metal ions (Fe^3+^, Ni^2+^, Cu^2+^, Mn^2+^, Ag^+^, Fe^2+^, Hg^2+^, Pd^2+^ and Cd^2+^) [41], which can be detected by the naked eye, implying that B-ANF can be used as a promising detector for metal ions in the future.

The filtration membrane plays an important role in industrial production and daily life. Good solvent resistance and mechanical strength are the necessary requirements for filtration membranes. ANF-based membranes with good mechanical properties and stability show great potential as filtration membranes. For example, when ANFs are used to coat the surface of a poly(ethylene terephthalate) (PET) non-woven membrane, the mechanical properties of the PET membranes can be improved and the composite membrane can work as an ultrafiltration membrane, realizing the removal of Au nanoparticles (10 nm) from solution with a rejection rate above 90% [42]. Additionally, ANFs can be incorporated into or mixed with many other polymers to prepare filtration membranes [43]. When ANFs were incorporated with silk fibers, the hybrid films showed an ultimate stress and Young’s modulus two times that of the pure silk film, enabling potential applications such as pressure-driven nanofiltration. By mixing PEI with ANF casting solution, a high-flux organic solvent nanofiltration membrane was fabricated by Bart et al. [44]. The membrane has a permeability more than 4 times higher than that of an ANF membrane. After 6 days of methanol treatment, the highest ethanol permeability was 9.1 L m^−2^h^−1^bar^−1^ and showed a high rejection rate of rose Bengal (RB).

### 2.4. Biomedical Application

The removal of metabolites and toxins in blood is still a major challenge for modern medicine. The problems of low efficiency, poor blood compatibility and easy pollution of blood purification materials need to be solved [45,46]. ANF composites have been used for blood purification because of their large surface area, controllable surface function, high porosity and great hydrophilicity. According to Zhao’s work [47], ANF beads prepared by dissolving micron-sized Kevlar fibers in proper solvent that showed good blood compatibility and low cytotoxicity, possessed an adsorption capacity higher than 40 mg g^−1^ towards bilirubin. Furthermore, beads with the addition of carbon nanotubes showed a stronger ability to adsorb human degradation waste.

**Figure 5:**
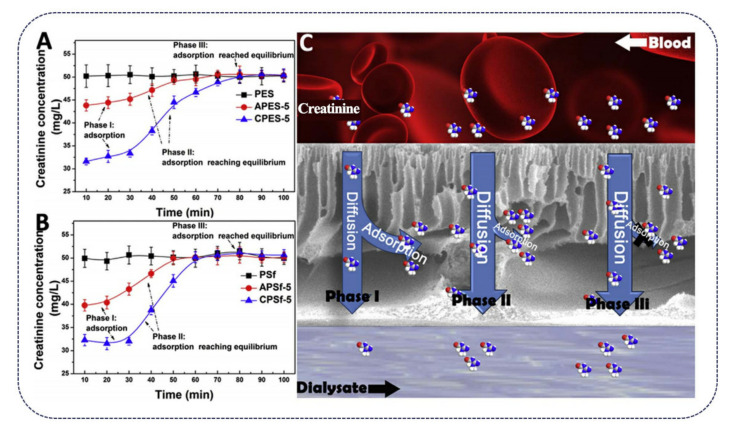
(**A**,**B**) Creatinine concentrations in the filtrate during the ultrafiltration by using PES- and PSF-based membranes, respectively. (**C**) Schematic image depicting the adsorption and diffusion of creatinine during ultrafiltration [48] (Copyright 2017, Elsevier).

ANFs are used to improve the biological and filtration properties of other polymer membranes. For example, polysulfon (PSF) and polyether sulfone (PES) are commonly used as ultrafiltration membranes in medicine, but they are often contaminated by the adhesion of proteins and organisms [48]. Due to the addition of ANFs, the membranes had higher water fluxes and retained high BSA rejection ratios compared to their pristine counterparts. ANF-modified membranes also show improved blood compatibility, and, furthermore, the blending of ANFs improved the efficiency of the composite membranes in removing creatinine toxins (Figure 5). In addition, ANFs have been shown to have potential in the development of load-bearing biomaterials [12]. The biomimetic hydrogels fabricated by aramid nanofibers interlacing with poly(vinyl alcohol), with water contents of as high as 70–92%, meanwhile, due to their excellent mechanical strength, match or exceed those of prototype tissues, e.g., cartilage.

### 2.5. Electromagnetics Applications

Compared with metallic materials, electrically conductive polymer composites, with the characteristics of lightweight resistance to corrosion and easy processing, have been widely used as electric heating materials [49]. Composites composed of ANFs and conductive materials usually show excellent abilities to convert electric energy into heat energy. For example, ANFs were used to fabricate nanocomposite papers with Ag nanowires (AgNWs) by vacuum-assisted filtration followed by a hot-pressing approach [50]. The heating temperature of electrical heaters containing 0.5 g/m^2^ AgNWs can be stabilized above 200 °C at 2.5 V. Furthermore, for materials with a conductive network, good conductivity is often accompanied by excellent electromagnetic interference (EMI) shielding performance. Examples include composites prepared by ANFs and CNT [51], polyaniline (PANI) [52] and Mxene [53]. For example, hydrated aramid nanofiber (HANF) can be mixed with expanded graphite (EG); moreover, 10 wt. % HANF can endow EG films with good flexibility, and the EMI shielding property of EG film reached 34.9 dB at the thickness of 30 μm [54].

As modern electrical equipment is gradually becoming miniaturized and high powered, dielectric materials with excellent mechanical properties and dielectric properties are indispensable [55]. ANFs were combined with some inorganic materials, such as montmorillonite [56] and BNNs [57], to prepare composites for electrical insulating material. A bioinspired ultra-tough multifunctional mica-based nanopaper containing a 3D aramid nanofiber framework has been reported [58]. The high dielectric strength (164 kV mm^−1^), excellent heat resistance (Tg = 268 °C) and nonflammability render the mica-based nanopaper a promising electrical insulating material in miniaturized high-power electrical equipment.

### 2.6. Other Applications

ANF composites can also be used for thermal insulation, heat conduction and heat dissipation. For example, ANFs can be directly made into aerogels with low density and low thermal conductivity, which can be further combined with phase change composites (PCM) to obtain ANF/PCM composites, such as the aerogel film with excellent thermal insulation performance that demonstrated high performance in military infrared stealth [59,60]. High temperature stability makes ANFs an ideal strengthening component of thermal conductivity composites. By combining them with BNNs [61,62], high-strength and high-thermal conductivity composites can be obtained, which can be used for heat dissipation in high-speed communication equipment and high-power devices. The combination of conducting polymers endowing ANFs with high electric conductivity and the responsivity of conductivity to different stimuli (human motions, spoken words and environmental humidity variation) means that ANF nanocomposites show great potential for applications in, for example, sensing garments, wearable hardware and rehabilitation [63].

## 3. Conclusions and Prospects

ANFs, as a new kind of nano building block, possess many advantages, such as excellent mechanical properties, high chemical corrosion resistance and thermal stability. In this short review, the applications of ANFs in high-strength composites, energy storage, filtration and adsorption, biomedical fields, amongst other fields, were briefly summarized, and some special properties of ANFs and their composites are listed in Table 1. Excluding the merits of mechanical properties, the prepared ANF composites also showed very good electrical conductivity, superior adsorption performance, EMI shielding and many other exciting properties, implying that ANFs will have a bright prospect in the next few years. However, the applications of ANFs are still in an early stage, and there is a long way to go before putting ANFs to practical use.

Due to their impressive properties, in the next few years, the research into ANFs is expected to attract an increasing amount of attention. It should be noted that the present method used to prepare ANFs is time consuming and cannot realize its production on a macro-scale. Thus, simple and efficient methods to prepare ANFs on such a scale should be proposed so as to enable wider applications. Secondly, phase compatibility is a critical factor for nanocomposites, and, thus, the surface treatment or surface tailoring of ANFs is also an interesting topic, exploration of which may greatly improve the properties of ANF composites. In addition, advanced functional materials being a goal of researchers, ANF-based functional materials for use in special fields make attractive research subjects, and we are sure that an increasing number of materials composed of ANFs will be developed and will be found to show potential applications in emerging fields, such as intelligent sensing, flexible wearable devices and biomedical fields.

## Figures and Tables

**Table 1:** Application of ANF composites.

General Application Fields	Other Composites Component	Practical Application Prospect	Key Properties	Ref.
High-strength nanocomposites	PVA	Fuel cells, water desalination units; batteries; filters	Water content (70–92%); tensile moduli ≈ 9.1 MPa; ultimate tensile strains ≈ 325%; compressive strengths ≈ 26 MPa	[12]
	PU	High-strength nanocomposites	Modulus (5.275 GPa); strength (98.02 MPa)	[13]
	PSU	Engineering plastics	Tensile strength (79 MPa)	[30]
Energy storage	PEO	Ion-conducting membranes	Ionic conductivity (1.7 × 10^−4^ S·cm^−1^)	[16]
	GO	Supercapacitor electrodes	Areal capacitance (221 μF/cm^2^)	[22]
	GO	Supercapacitor electrodes	Specific capacitance (128 F/g)	[23]
	PEO-LiTFSI	Solid polymer electrolytes	Conductivity (8.8 × 10^−5^ S·cm^−1^ at 30 °C; 1.0 × 10^−3^ S·cm^−1^ at 60 °C)	[34]
	PEDOT:PSS	Supercapacitor electrode	Electrical conductivity (534.2 S·cm^−1^); a specific capacitance (111.5 F/g at 0.5 A·cm^−3^)	[35]
	PANI	Supercapacitor electrode	Specific capacitance (441.0 F/g at 1 A/g)	[36]
Adsorption and filtration	MOF	Mechanically strong MOF hybrid aerogels	Superior adsorption performance for organic dyes; high flux (620 L/(h·m^2^·bar))	[40]
	Benzimidazole	Recyclable detector for heavy metal ions	Fast detection of heavy metal ions	[41]
	PEI	Organic solvent nanofiltration	Ethanol flux (9.1 L m^−2^ h^−1^ bar^−1^); high RB rejection of 98.4%	[44]
Biomedical application	PSF; PES	Water purification and clinical hemodialysis	Antifouling; good blood compatibility; remove creatinine toxin	[48]
Electromagnetics applications	AgNWs	Wearable devices; artificial intelligence	Sheet resistance (minimum Rs of 0.12 Ω/sq)	[50]
	CNT	Wearable electronics; EMI shielding clothing; personal thermal management systems	Electrical conductivity (230 S·m^−1^); EMI shielding property (54.4 dB at thickness of 568 μm)	[51]
	Mxene	Flexible electronic devices and wearable equipment	Specific EMI shielding property (8814.5 dB·cm^2^·g^−1^).	[53]
	EG	Smart phones and flexible electronic devices	Electrical conductivity (215 S·cm^−1^); EMI shielding property (34.9 dB, at thickness of 30 μm)	[54]
	Montmorillonite	Flexible electronics; high-voltage electrical insulation	Dielectric insulation performance (77.2 kV/mm)	[56]
	BNNS	Flexible electronics; energy storage; high-temperature electric power devices	Electrically insulating (>1015 Ω cm); thermally conductive (≈2.4 W·m^−1^·K^−1^); breakdown strength (≈292 MV·m^−1^).	[57]
Others	Phase-change materials	Infrared stealth	Thermal conductivity (0.036 W·m^−1^·K^−1^)	[60]
	Polypyrrole	Strain and humidity sensors	Electric conductivity up to ~25 S m^−1^	[63]

## Data Availability

The data presented in this study are available on request from the corresponding author.
